# Toll-like receptor homolog RP105 modulates the antigen-presenting cell function and regulates the development of collagen-induced arthritis

**DOI:** 10.1186/ar2529

**Published:** 2008-10-11

**Authors:** Yoshifumi Tada, Syuichi Koarada, Fumitaka Morito, Mio Mitamura, Hisako Inoue, Rie Suematsu, Akihide Ohta, Kensuke Miyake, Kohei Nagasawa

**Affiliations:** 1 Department of Internal Medicine, Saga Medical School, 5-1-1 Nabeshima, Saga 849-8501, Japan; 2 Department of Clinical Nursing, Saga Medical School, 5-1-1 Nabeshima, Saga 849-8501, Japan; 3 Division of Infectious Genetics, Institute of Medical Science, University of Tokyo, 4-6-1 Shirokanedai, Minato-ku, Tokyo 108-8639, Japan

## Abstract

**Introduction:**

RP105 is a Toll-like receptor homolog expressed on B cells, dendritic cells (DCs), and macrophages. We investigated the role of RP105 in the development of collagen-induced arthritis (CIA).

**Methods:**

CIA was induced in RP105-deficient DBA/1 mice and the incidence and arthritis index were analyzed. The cytokine production by spleen cells was determined. The functions of the DCs and regulatory T cells (Tregs) from RP105-deficient or control mice were determined by adding these cells to the lymph node cell culture. Arthritis was also induced by incomplete Freund's adjuvant (IFA) plus collagen or by injecting anti-collagen antibody and lipopolysaccharide.

**Results:**

RP105-deficient mice showed accelerated onset of arthritis and increased severity. Interferon-gamma (IFN-γ) and tumor necrosis factor-alpha production by spleen cells from RP105-deficient mice was increased in comparison with that from wild-type mice. The DCs from RP105-deficient mice induced more IFN-γ production, whereas Tregs from those mice showed less inhibitory effect against IFN-γ production. RP105-deficient mice also showed more severe arthritis induced by collagen with IFA.

**Conclusions:**

These results indicate that RP105 regulates the antigen-presenting cell function and Treg development, which induced the attenuation of the cell-mediated immune responses and, as a result, suppressed the development of CIA.

## Introduction

The Toll-like receptor (TLR) family is composed of pattern recognition receptors that recognize the pathogen-associated molecular patterns of microorganisms and trigger an innate immune response [1]. The TLRs are expressed mainly on macrophages and dendritic cells (DCs) and activate these cells after binding to their ligands. The activation of TLRs has been shown to induce proinflammatory cytokines, such as tumor necrosis factor-alpha (TNF-α) and interleukin-1 (IL-1), and also to cause the upregulation of costimulatory molecules which activates the adaptive immune system [2,3]. Whereas the TLRs play a key role for host defense, they also play important roles in inflammatory diseases [4]. Rheumatoid arthritis (RA) is a chronic autoimmune and inflammatory disease characterized by synovial inflammation and destruction of cartilage and bone. Recently, TLRs have been shown to play an important role in arthritis both in humans and in experimental animal models. In RA, it has been shown that TLR2, TLR3, TLR4, and TLR7 are upregulated in the synovium and synovial macrophages [5-9]. Some of these TLRs are upregulated by proinflammatory cytokines and, in turn, have a costimulatory function [7,8]. The endogenous ligands of TLRs, such as heat shock proteins [10-12], hyaluronan [13,14], or degradation product of fibrinogen [15], are expressed in joints, and it is considered that they can activate DCs or macrophages via the TLR, thus leading to the progression of arthritis in joints [16,17].

In experimental animal models, the critical roles of the TLRs and their adaptor molecule myeloid differentiation factor 88 (MyD88) in the development of arthritis have been demonstrated in various models [18-22]. The data that the injection of TLR3 and TLR9 ligands into the joints induced arthritis [23,24] and that TLR9 ligand CpG immunization induces arthritis in rats [25] further support an arthritogenic role of TLRs. On the contrary, systemic TLR3 activation has been shown to suppress antibody-induced and TCR-transgenic mouse serum-induced arthritis [26], thus suggesting the different effect in arthritis between the local and systemic activation of TLRs.

RP105, expressed on B cells, macrophages, and DCs, is a TLR homolog that lacks a conserved intracellular signaling domain (Toll-IL-1 receptor domain) and forms a complex with soluble protein MD-1 [27-29]. It has been shown that RP105 can provide proliferation and activation signals in B cells [28] and that B cells from RP105-deficient mice were hyporesponsive to TLR4 and TLR2 stimulation [30,31]. We have been working on RP105 on B cells in patients with autoimmune diseases, including systemic lupus erythematosus. We previously reported that B cells lacking RP105 expand in the peripheral blood of patients with systemic lupus erythematosus [32] and that these cells can produce anti-double-stranded DNA antibody [33]. On the other hand, Divanovic and colleagues [34] showed that RP105 directly interacts with TLR4 and negatively regulates TLR4 signaling by experiments using transfectant cells and RP105-deficient mice-derived DCs.

In the present study, we investigated the role of RP105 in the development of collagen-induced arthritis (CIA) using RP105-deficient mice. CIA is an autoimmune inflammatory disease of the joints which is induced by immunization with type II collagen (CII). Our data show that RP105-deficient mice exhibit an accelerated onset of more severe arthritis, with an increased cytokine production of T cells and attenuated development of regulatory T cells (Tregs). These results suggest that RP105 plays a regulatory role in cell-mediated immunity and the development of CIA.

## Materials and methods

### Mice and experimental conditions

RP105-deficient mice [30] were backcrossed into the DBA/1 background for six generations and genotyped by polymerase chain reaction using ear biopsy-derived DNA. In all experiments, only RP105^-/-^, RP105^+/-^, and RP105^+/+ ^littermates were used. All mice were 12 to 16 weeks of age at the time of immunization. The animals were maintained at the Saga Medical School animal facility. The care of the animals was in accordance with the guidelines for animal experimentation of the Saga Medical School. All animal experiments were approved by the local ethical committee (University of Saga, numbers 07-015-4 and 07-015-5).

### Induction of collagen-induced arthritis

Mice were immunized intradermally at the base of the tail with 150 μg of bovine CII (Cosmo Bio Co. Ltd., Tokyo, Japan) emulsified with an equal volume of complete Freund's adjuvant (CFA) containing 200 μg of H37RA *Mycobacterium tuberculosis *(Difco Laboratories Inc., now part of Becton Dickinson and Company, Franklin Lakes, NJ, USA) on day 0. Mice were boosted by an intradermal injection of 150 μg of bovine CII in incomplete Freund's adjuvant (IFA) (Difco Laboratories Inc) on day 21. Arthritis development was monitored by physical examination three times per week and the inflammation in each of the four paws was graded from 0 to 3, as described previously [35]. The four scores were added, with the maximum score per mouse being 12. The arthritis index was calculated by dividing the total score of the experimental mice by the number of total mice or arthritic mice. In the additional experiment, CIA was induced without CFA; the mice were immunized with 200 μg of bovine CII emulsified with IFA on days 0 and 21.

### Histology

The ankle joints of the mice were excised 5 weeks after immunization and fixed in 10% buffered formalin, decalcified in 10% EDTA (ethylenediaminetetraacetic acid), embedded in paraffin, sectioned, and stained with hematoxylin and eosin. The intensity of synovial hyperplasia, cellular infiltration, and pannus formation was examined and arthritis was graded in a blinded fashion on a scale of 0 to 4, as described previously [36].

### Measurement of the serum anti-CII antibody levels

The levels of serum antibodies to CII were measured by enzyme-linked immunosorbent assay (ELISA) as previously described [37]. Briefly, serial dilutions of serum samples were added to the microtiter plates (Maxisorp; Nunc, Roskilde, Denmark), coated with native bovine CII at 10 μg/mL, and incubated for 1 hour at 37°C. After washing, peroxidase-conjugated goat anti-mouse IgG1 or IgG2a (SouthernBiotech, Birmingham, AL, USA) was added and incubated for 1 hour at 37°C. Antibody binding was visualized using orthophenylenediamine (Sigma-Aldrich, St. Louis, MO, USA). A standard serum composed of a mixture of sera from the arthritic mice was added to each plate in serial dilutions and a standard curve was constructed. The standard serum was defined as 100 U and antibody titers of serum samples were calculated from the standard curve.

### Measurement of cytokine production by spleen cells

Interferon-gamma (IFN-γ), TNF-α, and IL-2 and IL-4 production was examined on day 28 in the spleen cells. The cells were resuspended in Dulbecco's modified Eagle's medium (DMEM) with 2% autologous mouse serum, seeded at 6 × 10^6 ^per wells in 24-well plates (Nunc), and stimulated with denatured CII (dCII) or Con A (Sigma-Aldrich) for 48 hours. The cytokines produced in the culture supernatant were measured by the cytometric beads assay system (BD Biosciences, San Jose, CA, USA) using FACSCalibur, except for the measurement of IL-17, which was done by ELISA (R&D Systems, Minneapolis, MN, USA).

### Preparation and functional analysis of splenic dendritic cells

The splenic DCs were purified by magnetic cell sorting using CD11c microbeads (Miltenyi Biotec, Auburn, CA, USA) in accordance with the manufacturer's instructions. Briefly, the spleens from two or three mice immunized with CII 8 days before were digested with collagenase D (Roche Diagnostics, Penzberg, Germany). The spleen cells were incubated with anti-CD16/32 antibody for blocking, labeled with CD11c magnetic-activated cell sorting beads, and applied to magnetic column. After washing the column, the CD11c^+ ^DCs were obtained by flushing them out using a plunger. The resultant cell purity was greater than 92% by flow cytometry. The conventional DCs (cDCs) and plasmacytoid DCs (pDCs) were determined by staining with anti-CD11c plus anti-B220 and anti-PDCA-1 plus anti-B220 (Miltenyi Biotec). For functional analysis, the splenic DCs (50 or 12.5 × 10^4 ^cells) from RP105^+/+ ^or RP105^-/- ^mice were mixed with pooled adherent cell-removed lymph node cells (LNCs) (2.5 × 10^6 ^cells) from RP105^+/- ^mice, which were immunized with CII 8 days before. The cells were resuspended in DMEM with 2% mouse serum and stimulated with dCII for 3 days. The IFN-γ produced in the culture supernatant was measured using ELISA kits (Biosource International, Camarillo, CA, USA).

### Measurement of cytokine production by splenic dendritic cells

The splenic DCs from RP105^+/+ ^and RP105^-/- ^mice separated as described were resuspended at 4 × 10^5 ^per well in 96-well microtiter plates and stimulated with various concentrations of lipopolysaccharide (LPS) (OB111; Sigma-Aldrich) for 24 hours. The cytokines produced in the culture supernatant were measured by the cytometric beads assay system (BD Biosciences) using FACSCalibur. The lower detection limit of cytokines was 3.0 pg/mL in these experiments.

### Preparation and functional analysis of regulatory T cells

The Tregs were purified from the spleens by magnetic cell sorting using the CD4^+^CD25^+ ^regulatory T-cell isolation kit (Miltenyi Biotec) in accordance with the manufacturer's instructions. Briefly, the spleens from three mice, immunized with CII 4 weeks before, were digested with collagenase D (Roche Diagnostics). The spleen cells were incubated with a biotin-labeled antibody cocktail and further incubated with anti-biotin microbeads and phycoerythrin-labeled anti-CD25 antibody. After washing, the cells were applied to the LD (lymphocyte depletion) column, and the T cells that passed through the column were collected. Thereafter, the T cells were incubated with anti-phycoerythrin microbeads, washed, and applied to the LS (lymphocyte separation) column. After washing the column, the CD4^+^CD25^+ ^T cells were obtained by flushing them out with a plunger. The resultant cell purity was greater than 90% by flow cytometry, and the contaminating cells were mainly non-T non-B cells. Foxp3 staining was performed to compare the purity and its expression in recovered cells as well as whole spleen cells using fluorescein isothiocyanate-conjugated anti-Foxp3 antibody (eBioscience, San Diego, CA, USA). More than 95% of the recovered CD4^+^CD25^+ ^T cells were positive for Foxp3, which was not different between cells from RP105^+/+ ^or RP105^-/- ^mice. For a functional analysis, Tregs (1.0 × 10^5 ^cells) from RP105^+/+ ^or RP105^-/- ^mice were added to the pooled LNCs (1.0 × 10^6 ^cells) from RP105^+/- ^mice, which were immunized with CII 8 days before, resuspended in DMEM with 2% mouse sera, and stimulated with dCII or anti-CD3 antibody (BD Biosciences) for 3 days. The IFN-γ produced in the culture supernatant was measured using ELISA kits (Biosource International).

### Induction of anti-CII antibody-induced arthritis

Anti-CII antibody-induced arthritis was induced using a kit (Chondrex Inc., Redmond, WA, USA). RP105^+/+ ^and RP105^-/- ^mice were intravenously injected with 2 mg of a cocktail of monoclonal anti-CII antibodies. Three days later, 10 or 50 μg of LPS (Sigma-Aldrich) was injected intraperitoneally, and the mice were examined daily for signs of arthritis. In addition, the serum TNF-α levels after LPS challenge were determined. One hour after an intraperitoneal injection of LPS, the sera were collected and the TNF-α levels were measured using ELISA kits (Biosource International).

### Statistical analysis

The analyses of the incidence of arthritis were performed using the chi-square analysis. The significance of the differences in the arthritic indexes, anti-CII antibody levels, and cytokine production was determined using either the Mann-Whitney *U *test or the Wilcoxon matched pairs test.

## Results

**Figure 1:**
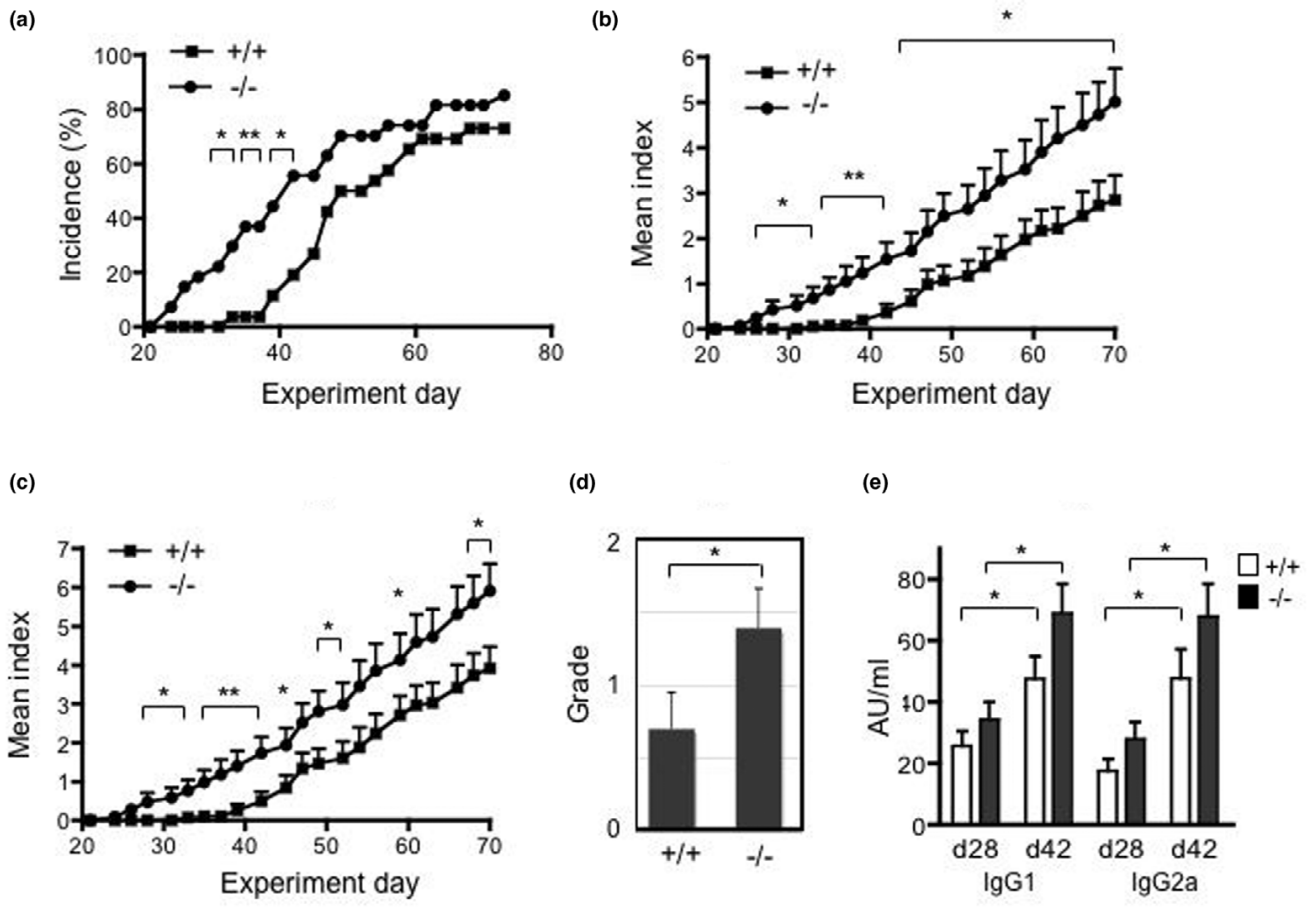
The development of collagen-induced arthritis in RP105-deficient mice. **(a-d) **RP105^+/+ ^(n = 26, 13 males and 13 females) and RP105^-/- ^(n = 27, 13 males and 14 females) mice were immunized with type II collagen (CII), and signs of arthritis were monitored as described in Materials and methods. The incidence of arthritis in RP105^-/- ^mice was higher than that in RP105^+/+ ^mice between days 31 and 42 (*P < 0.05 and **P < 0.01 for comparison with RP105^+/+ ^mice, chi-square test) **(a)**. The disease severity, expressed as the mean arthritis index (and standard error) of the total mice **(b)** and of the arthritic mice **(c)**, is shown (*P < 0.05 and **P < 0.01 for comparison with RP105^+/+ ^mice, Mann-Whitney *U *test). **(d)** A histological examination was performed in RP105^+/+ ^(n = 20, 10 males and 10 females) and RP105^-/- ^(n = 23, 11 males and 12 females) mice at day 35 (*P < 0.05, chi-square test). **(e) **The anti-CII antibodies of IgG1 and IgG2a classes were measured from RP105^+/+ ^(n = 18) and RP105^-/- ^(n = 19) mice on days 28 and 42. Values are mean ± standard error (*P < 0.001, Wilcoxon matched pairs test). AU, arbitrary units.

RP105-deficient mice developed more severe collagen-induced arthritisTo examine the role of RP105 in the development of CIA, RP105^+/+ ^and RP105^-/- ^littermate mice were immunized with CII and monitored for signs of arthritis. As shown in Figure 1a, RP105^-/- ^mice showed an earlier onset of disease, and a significantly higher incidence was observed from day 31 to day 42 than in RP105^+/+ ^mice. After that period, the incidence of arthritis in RP105^+/+ ^mice increased and no more differences were observed. The final rates of incidence of arthritis were 85.2% and 73.1% in RP105^-/- ^and RP105^+/+ ^mice, respectively. As shown in Figure 1b and 1c, the arthritis index increased gradually, thus indicating the chronic progression of the disease in a similar manner; however, the arthritis index in RP105^-/- ^mice was significantly higher than that in RP105^+/+ ^mice. To histologically determine the grade of arthritis, the ankle joints were excised, and the degree of cellular infiltration and synovial hyperplasia was examined. As shown in Figure 1d, the ankle joints of RP105^-/- ^mice showed more severe synovitis, especially more inflammatory cell infiltration, than those of RP105^+/+ ^mice at an early stage (day 35). We also compared the histology of arthritis at a late stage (7 to 8 weeks). Although RP105^-/- ^mice showed a relatively severe degree of arthritis, the difference was not statistically significant (data not shown). To assess the humoral immune response to CII in RP105-deficient mice, the IgG1 and IgG2a anti-CII antibody levels in the sera of mice were determined on days 28 and 42. As shown in Figure 1e, although the anti-CII antibodies of both IgG subclasses seemed to be slightly higher in RP105^-/- ^mice, they were not significantly different. The anti-CII antibody levels increased from day 28 to day 42 in RP105^+/+ ^and RP105^-/- ^mice. These results indicate that RP105^-/- ^mice showed an accelerated onset of CIA and a more severe CIA and suggest that the RP105 molecule may therefore regulate the development of CIA.

### Interferon-gamma and tumor necrosis factor-alpha production by spleen cells was augmented in RP105-deficient mice

**Figure 2:**
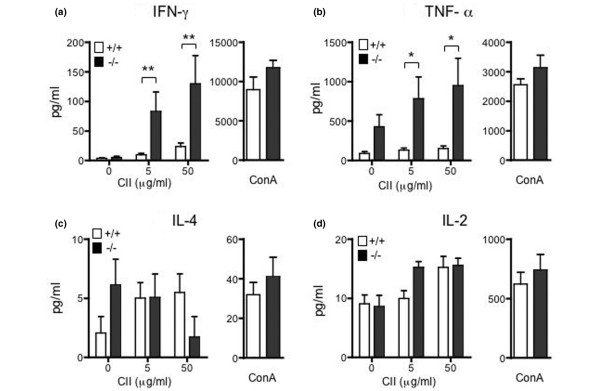
The cytokine production from spleen cells in response to denatured type II collagen (CII) and ConA in RP105-deficient mice. Mice were immunized with CII with complete Freund's adjuvant on day 0 and with CII with incomplete Freund's adjuvant on day 21. The spleen cells from day-28 mice were harvested and stimulated with CII or ConA (5 μg/mL) for 48 hours. Interferon-gamma (IFN-γ) **(a)**, tumor necrosis factor-alpha (TNF-α) **(b)**, interleukin-4 (IL-4) **(c)**, and interleukin-2 (IL-2) **(d) **produced in the culture supernatant were measured by cytometric beads assay. The results from two experiments (eight mice per experiment) are shown and expressed as the mean ± standard error (*P < 0.05, **P < 0.01, Mann-Whitney *U *test).

To analyze the antigen-specific responses of the spleen cells, the production of cytokines in response to CII was examined. CII-immunized mice were sacrificed on day 28, and the splenocytes were stimulated with dCII or ConA. As shown in Figure 2a and 2b, the spleen cells from RP105^-/- ^mice produced a markedly larger amount of IFN-γ and TNF-α in response to CII than did RP105^+/+ ^mice (*P *< 0.01 and *P *< 0.05, respectively), but not in response to ConA. The production of IL-2 and IL-4 was not significantly different between RP105^+/+ ^and RP105^-/- ^mice (Figure 2c, d). In addition, IL-17 production from spleen cells in response to CII (50 μg/dL) was similar in RP105^+/+ ^and RP105^-/- ^mice (781.8 ± 167.3 pg/mL and 840.3 ± 236.0 pg/mL, n = 5).

### Dendritic cells from RP105-deficient mice induced more interferon-gamma production from lymph node cells than dendritic cells from wild-type mice

**Figure 3:**
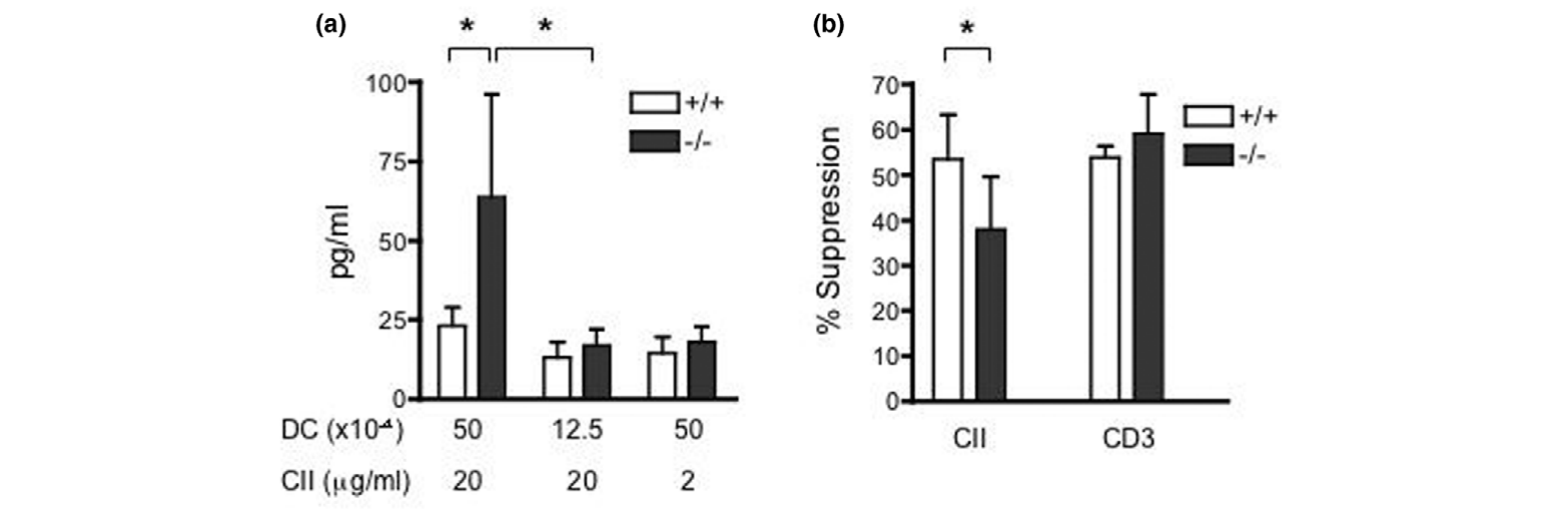
The functional analysis of dendritic cells (DCs) and regulatory T cells (Tregs) from RP105-deficient mice. **(a) **The splenic DCs from type II collagen (CII)-immunized RP105^+/+ ^or RP105^-/- ^mice were mixed with pooled adherent cells removed lymph node cells (LNCs) from RP105^+/- ^mice, as described in Materials and methods. The cells were stimulated with denatured CII for 2 days and interferon-gamma (IFN-γ) production was measured. Values are mean ± standard error. The DCs from RP105^-/- ^mice induced higher IFN-γ production from LNCs than did the DCs from RP105^+/+ ^at 50 × 10^4 ^cells. The summary of five experiments, using three mice per group, is shown (*P < 0.05, Mann-Whitney *U *test). **(b) **The Tregs (1 × 10^5^) purified from the spleens of CII-immunized RP105^+/+ ^and RP105^-/- ^mice were mixed with pooled LNCs (1 × 10^6^) from RP105^+/- ^mice, which were immunized with CII 14 days before. The cells were stimulated with denatured CII (20 μg/mL) or anti-CD3 antibody (1 μg/mL) for 2 days and IFN-γ production was measured. The percentage suppression (100 × [IFN-γ without Tregs – IFN-γ with Tregs]/IFN-γ without Tregs) is shown. Values are mean ± standard error of the six experiments, using three mice per group (*P < 0.05, Wilcoxon matched pairs test).

**Figure 4:**
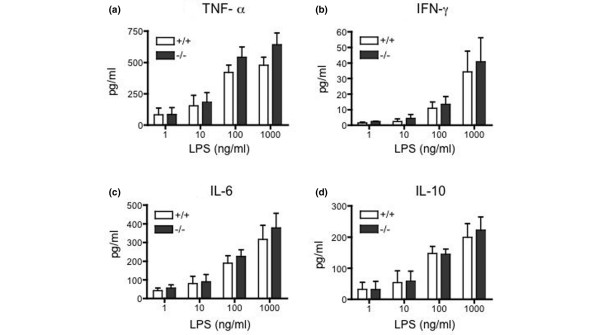
The inflammatory cytokine production from splenic dendritic cells in response to lipopolysaccharide (LPS) in RP105-deficient mice. The dendritic cells (4 × 10^5^) purified from the spleens of RP105^+/+ ^and RP105^-/- ^mice were stimulated with LPS at various concentrations for 24 hours. The supernatants were collected and the concentrations of tumor necrosis factor-alpha (TNF-α) **(a)**, interferon-gamma (IFN-γ) **(b)**, interleukin-6 (IL-6) **(c)**, and interleukin-10 (IL-10) **(d) **were measured. Values are mean ± standard error of five experiments, using three mice per group.

Because RP105 is not expressed on T cells, we speculated that the augmented cytokine production by spleen cells from RP105-deficient mice would be driven by antigen-presenting cells (APCs). To compare the function of DCs, the splenic DCs were purified from the CII-immunized RP105^+/+ ^or RP105^-/- ^mice, mixed with adherent cell-depleted LNCs from CII-immunized RP105^+/- ^mice, and stimulated with dCII. As shown in Figure 3a, RP105^-/- ^splenic DCs induced higher amounts of IFN-γ by LNCs than RP105^+/+ ^DCs at the higher cell numbers. Prepared splenic DCs consisted of greater than 70% cDCs (CD11c^+^, B220^-^) and less than 30% pDCs (PDCA-1^+^, B220^+^), and the subpopulation did not differ between RP105^+/+ ^and RP105^-/- ^mice (cDCs: 76% ± 5% versus 75% ± 2%). To evaluate DC function, we measured the cytokine production from DCs in response to LPS. The splenic DCs from the two types of mice produced similar amounts of TNF-α, IFN-γ, and IL-6 and IL-10 in response to various concentrations of LPS (Figure 4).

### Reduced suppressive function of regulatory T cells in response to CII but not to anti-CD3 from RP105-deficient mice

We investigated the function of Tregs from RP105-deficient mice because a recent report showed that the expression of MD1, a molecule that forms a complex with RP105, regulated the development of Tregs induced by LPS [38]. We first measured the number of Tregs in the spleen by flow cytometry. The percentages of CD4^+^CD25^+ ^T cells among spleen cells and of CD25^+ ^cells among CD4^+ ^T cells were not different between RP105^+/+ ^and RP105^-/- ^mice (1.47% ± 0.13% versus 1.33% ± 0.18% and 11.6% ± 0.9% versus 11.7% ± 2.0%, respectively, n = 5). Similarly, the population of Foxp3-positive CD4^+ ^T cells was not different (RP105^+/+ ^1.47% ± 0.20% and RP105^-/- ^1.40% ± 0.16%). Next, we examined the suppressive function of Tregs by measuring the IFN-γ production by LNCs with or without the addition of Tregs. Tregs from the spleens of CII-immunized RP105^+/+ ^or RP105^-/- ^mice were mixed with LNCs from CII-immunized RP105^+/- ^mice and stimulated with dCII or anti-CD3 antibody. From the preliminary experiment, we chose the LNC/Treg ratio for which the suppression of IFN-γ production reached plateau level. As shown in Figure 3b, although Tregs suppressed the IFN-γ production from LNCs, the Tregs from RP105^-/- ^mice showed less suppression than those from RP105^+/+ ^mice when they were stimulated with dCII. On the other hand, stimulation with anti-CD3 antibody induced comparable levels of suppression by Tregs from RP105^+/+ ^and RP105^-/- ^mice (Figure 3b). These results indicate that RP105^-/- ^mice have attenuated antigen-induced suppressive function of Tregs or had a decreased number of CII-specific Tregs.

### RP105-deficient mice showed marginally augmented disease development in anti-CII antibody and lipopolysaccharide-induced arthritis

**Figure 5:**
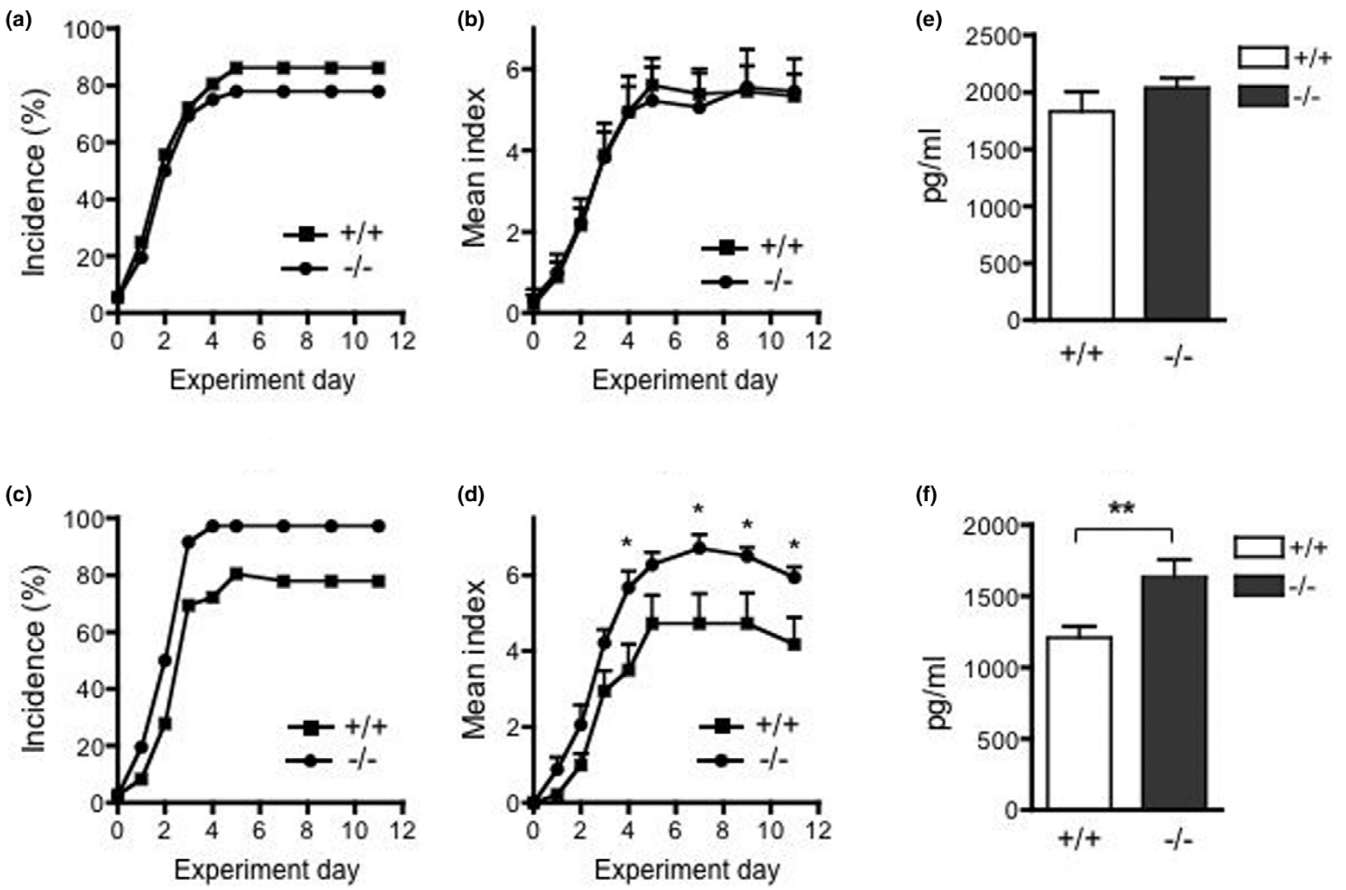
The development of anti-CII antibody and lipopolysaccharide (LPS)-induced arthritis and tumor necrosis factor-alpha production after LPS challenge in RP105-deficient mice. **(a-d) **RP105^+/+ ^(n = 9) and RP105^-/- ^(n = 9) mice were injected with a cocktail of monoclonal anti-CII antibodies intravenously and with 50 μg (a, b) or 10 μg (c, d) of LPS intraperitoneally 2 days later. The incidence of arthritis of paws (arthritic paws/total paws) (a, c) and disease severity, expressed as the mean arthritis index (and standard error) of mice (b, d), is shown. Arthritis index was significantly higher in RP105^-/- ^mice with 10 μg of LPS (*P < 0.05 for comparison with RP105^+/+ ^mice, Mann-Whitney *U *test). **(e, f) **RP105^+/+ ^and RP105^-/- ^mice were challenged intraperitoneally with 50 μg (e) (n = 10 and 11, respectively) or 10 μg (f) (n = 7 and 9, respectively) of LPS. The serum was collected 1 hour later and the tumor necrosis factor-alpha levels were measured. Values are mean ± standard error. **P < 0.01, Mann-Whitney *U *test. CII, type II collagen.

To investigate the development of arthritis induced by LPS in RP105-deficient mice, we injected a cocktail of monoclonal anti-CII antibodies intravenously followed by the intraperitoneal injection of LPS. As shown in Figure 5a and 5b, RP105^+/+ ^and RP105^-/- ^mice showed similar development of arthritis after 2 mg of anti-CII antibodies followed by 50 μg of LPS 3 days later. The arthritis index did not differ between RP105^+/+ ^and RP105^-/- ^mice. We then examined the induction of arthritis with a decreased dose of LPS to test the response to LPS more concisely. Whereas the reduction of the dose of LPS to 10 μg decreased the severity of arthritis in RP105^+/+ ^mice, RP105^-/- ^mice still developed arthritis with a comparable severity as induced by 50 μg of LPS (Figure 5c, d). We compared the production of TNF-α after the intraperitoneal administration of LPS in RP105^+/+ ^and RP105^-/- ^mice. Although the two types of mice produced similar levels of TNF-α in response to 50 μg of LPS (Figure 5e), RP105^-/- ^mice produced a significantly higher amount of TNF-α in response to 10 μg of LPS (Figure 5f). These data indicate that RP105^-/- ^mice showed an enhanced production of TNF-α and arthritis development with a lower dose of LPS, suggesting a regulatory role of RP105 in the *in vivo *response to LPS.

### RP105-deficient mice developed more severe arthritis induced by incomplete Freund's adjuvant and CII

**Figure 6:**
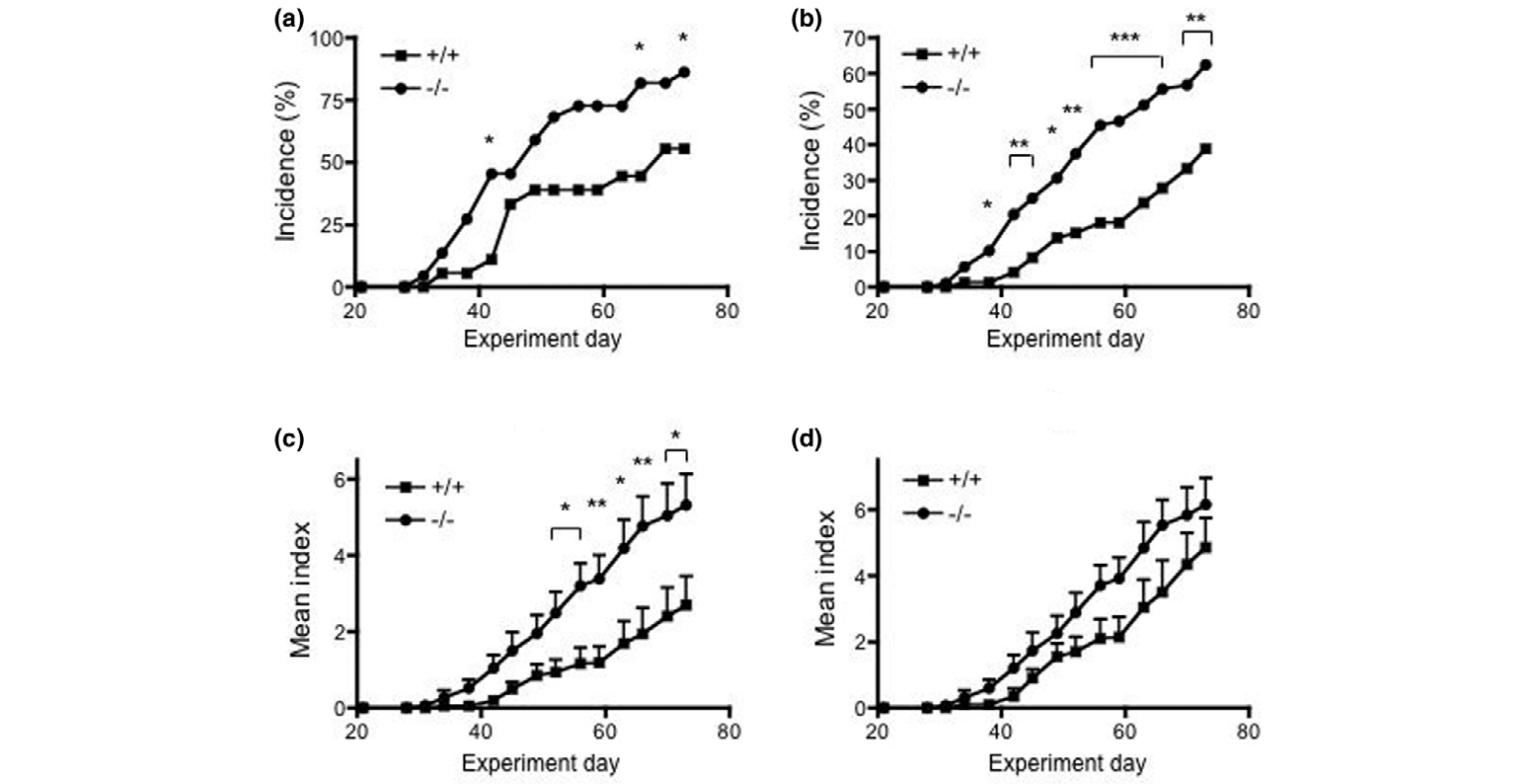
The development of collagen-induced arthritis induced with type II collagen (CII) and incomplete Freund's adjuvant (IFA) in RP105-deficient mice. RP105^+/+ ^(n = 18, 11 males and 7 females) and RP105^-/- ^(n = 22, 11 males and 11 females) mice were immunized with CII and IFA on days 0 and 21, and signs of arthritis were monitored. The arthritis incidence was shown as per body **(a) **and per paw (100 × [number of the arthritic paws/number of total paws]) **(b)**. The incidence of arthritis in RP105^-/- ^mice was higher than that in RP105^+/+ ^mice on days 42, 66, and 73 **(a)** as was the per-paw incidence after day 38 **(b)**. (*P < 0.05, **P < 0.01, and ***P < 0.001 for comparison with RP105^+/+ ^mice, chi-square test) **(c, d) **The disease severity, expressed as the mean arthritis index (and standard error) of total mice **(c)** and of arthritic mice **(d)**, is shown (*P < 0.05 and **P < 0.01 for comparison with RP105^+/+ ^mice, Mann-Whitney *U *test).

We addressed the question of whether the enhanced arthritis development in RP105^-/- ^mice was due to the enhanced response of TLRs to the *Mycobacterium*-containing adjuvant, CFA, because it has been shown that the *in vivo *and *in vitro *response to *Mycobacterium tuberculosis *is regulated by TLR2, TLR 4, and MyD88 [39-42]. To investigate the impact of the *Mycobacterium*-TLR interaction in the augmented arthritis development in RP105-deficient mice, we injected CII emulsified with IFA twice into RP105^+/+ ^and RP105^-/- ^mice. As shown in Figure 6, RP105^-/- ^mice still showed a higher incidence of arthritis and more severe disease development, although they were not as prominent as those observed in CIA with the regular CFA-based induction. These data indicate that the augmented arthritis development in RP105^-/- ^mice is not dependent solely on the CFA and suggest that other TLR ligands, presumably endogenous, might also play a role in this phenomenon.

## Discussion

In the present study, we showed augmented arthritis development in RP105^-/- ^mice, with an increased production of IFN-γ and TNF-α from spleen cells in response to CII. These results indicate that cell-mediated immune responses to immunized collagen are enhanced in RP105-deficient mice, thus suggesting RP105 to be a negative regulator of the immune system in this model.

RP105 was originally found as a molecule that induces B-cell activation after its ligation, but it has been shown to be expressed also on macrophages and DCs. The role of RP105 on these cells, however, has not yet been clearly determined. Recently, Divanovic and colleagues [34] showed that the expression of RP105 by gene transfection suppressed TLR4 signaling in HEK293 cells and that DCs and the macrophages from RP105^-/- ^mice produced a higher amount of cytokines in response to LPS. These data indicate that RP105 is an attenuator of TLR4-induced signaling [34]. We demonstrated here that DCs from RP105-deficient mice induced a larger amount of IFN-γ production from LNCs (Figure 3a), thus indicating the augmented T-cell-activating function of DCs lacking RP105. On the other hand, we did not see any significant differences between DCs from RP105-deficient mice and those from control mice in cytokine production in response to LPS (Figure 4). These data are in contrast to those of Divanovic and colleagues [34]. The difference might be due to the purity of the LPS because the conventional LPS that we used might have been contaminated with a lipopeptide, the TLR2 ligand [34]. Alternatively, the different DC preparation, splenic DCs versus bone marrow-derived DCs, may have produced different results.

In the anti-CII antibody and LPS-induced arthritis model, arthritis development and TNF-α production were enhanced in RP105-deficient mice when a reduced dose of LPS was administered. Although the difference in the arthritis index between wild-type and RP105-deficient mice was small in comparison with that when they were actively immunized, these results nevertheless support a regulatory role of RP105 in the development of arthritis induced by TLR ligands. On the other hand, these *in vivo *results cannot be explained by the *in vitro *data of splenic DCs (Figure 4). We therefore need to conduct more experiments using specific ligands and cells other than DCs, such as macrophages or synovial cells, to solve these problems.

TLRs have been shown to play a role in the acquired immune responses by promoting cytokine and chemokine production and costimulatory molecule expression. In addition, a recent report showed that MD1, a molecule that forms a complex with RP105, regulated the development of Tregs induced by LPS [38]. We also observed the impaired Treg function in RP105-deficient mice. The Tregs play key roles in the maintenance of immunologic self-tolerance and negative control of a variety of physiological and pathological immune responses [43,44]. The administration of a Treg-depleting anti-CD25 antibody induced an accelerated onset of arthritis and a more severe arthritis in murine CIA [45,46]. In our experiment, the suppressive function of RP105^-/- ^Tregs was impaired when the cells were stimulated with collagen, but not with anti-CD3 antibody. These data suggest that reduced Treg function is not intrinsic but instead is due to insufficient activation when triggered by antigen presented by APCs. Alternatively, the number of CII-specific Tregs is decreased in RP105-deficient mice. It has been shown that the suppressive effect of Tregs is blocked by DCs activated by TLR ligands, such as LPS or CpG, via IL-6 production [47]. Therefore, it is possible that impaired antigen-specific Treg induction in RP105-deficient mice is due to an overproduction of inflammatory cytokines by APCs activated by immunization with CII and CFA.

Next, we tried to elucidate whether the regulatory role of RP105 in the development of arthritis is exerted only when the disease was induced by active immunization using CFA. If enhanced arthritis development in RP105-deficient mice is CFA-dependent, it can be speculated that the activation signal through TLRs evoked by CFA, which contains heat-killed mycobacteria, is augmented in APCs lacking RP105. However, RP105-deficient mice showed a higher incidence even when arthritis was induced using IFA only, thus indicating that the enhancing effect in arthritis development does not depend solely on CFA. To explain these results, we hypothesized that the signals provided by endogenous TLR ligands other than CFA also made the difference between RP105-deficient and wild-type mice. Recently, various endogenous ligands of TLR4 and TLR2 have been reported. These molecules include fibrinogen degradation products [15], hyaluronan fragments [13,14], fibronectin fragments [48], heparan sulfate [49], high-mobility group box-1 protein [50], and heat shock proteins [10-12]. These molecules have been shown to induce the activation or the release of proinflammatory cytokines from DCs or macrophages. In addition, heat shock proteins, fibrinogen, and degraded hyaluronan are commonly found in inflamed joints. Therefore, it is possible that DCs and synovial macrophages lacking RP105 might be highly activated by these endogenous ligands, thus resulting in the enhanced development of arthritis. However, this hypothesis has yet to be investigated.

This study has a limitation regarding the specificity of TLRs. Because we used conventional LPS, which might have been contaminated with TLR2 ligands, we could not determine the precise role of TLR4 in our studies. As such, it remains to be investigated which TLR is regulated by RP105 in these models using more specific ligands and whether the *in vitro *activation and cytokine production from various cells (for example, macrophages and synovial cells) are altered in the absence of RP105.

RP105 is also expressed in B cells. B cells from RP105-deficient mice are hyporesponsive to LPS, in contrast to DCs, and to lipoproteins [30,31]. Our observation that RP105-deficient mice developed severe arthritis in response to anti-CII antibody and LPS and produced more TNF-α in response to LPS indicated that the hyporesponsiveness of RP105^-/- ^B cells might be overcome by other cells *in vivo*. In addition, RP105-deficient mice were not impaired with respect to T-cell-dependent antibody production [30]. In keeping with this, we observed that RP105^-/- ^mice showed comparable levels of anti-CII antibody in CIA.

Several studies have demonstrated the involvement of TLRs in arthritis models. The intra-articular triggering of TLRs led to the joint inflammation [23,24], and TLR2 and TLR4 have been shown to play a critical role in bacterial cell wall-induced arthritis [18,20]. In human RA, TLRs, including TLR2 and TLR4, are expressed in synovial cells and are upregulated by proinflammatory cytokines [5-9]. RA synovial cells have been shown to produce various chemokines after the stimulation with TLR2 ligands [51] and inflammatory cytokines after triggering TLR2 or TLR4 [52]. In addition, the suppression of MyD88 or Mal/TIRAP by transfecting the dominant negative form downregulated the production of cytokines and matrix metalloproteinases from synovial cells [52]. Although these studies emphasize the proinflammatory roles of TLRs and adaptor molecules, the regulatory or anti-inflammatory role of TLR family members is not known. Our data suggest that a potential regulatory mechanism exists in the TLR-TLR ligand system in inflammation.

## Conclusions

Our study demonstrated that RP105 regulated the cell-mediated immune response by the suppression of APC function and regulatory T-cell development and, as a result, attenuated the development of CIA. Further study is necessary to address the precise interaction between RP105 and various TLRs and the mechanism of regulation in the inflammatory process in the development of arthritis.

## Abbreviations

APC: antigen-presenting cell; cDC: conventional dendritic cell; CFA: complete Freund's adjuvant; CIA: collagen-induced arthritis; CII: type II collagen; DC: dendritic cell; dCII: denatured type II collagen; DMEM: Dulbecco's modified Eagle's medium; ELISA: enzyme-linked immunosorbent assay; IFA: incomplete Freund's adjuvant; IFN-γ: interferon-gamma; IL: interleukin; LNC: lymph node cell; LPS: lipopolysaccharide; MyD88: myeloid differentiation factor 88; pDC: plasmacytoid dendritic cell; RA: rheumatoid arthritis; TLR: Toll-like receptor; TNF-α: tumor necrosis factor-alpha; Treg: regulatory T cell.

## Competing interests

The authors declare that they have no competing interests.

## Authors' contributions

YT carried out the *in vivo *and *in vitro *studies. SK carried out the flow cytometric analysis. FM, AO, and KN participated in the design and coordination of the study. MM, HI, and RS participated in the design of the study and the maintenance of the animals. KM provided the animals and gave final approval of the version to be published. All authors read and approved the final manuscript.
